# PW03-005 - NLRP3-Q705K monocytes do not produce more IL-1B

**DOI:** 10.1186/1546-0096-11-S1-A231

**Published:** 2013-11-08

**Authors:** G Simon, N Busso, A von Scheven-Gete, I Moix, M Morris, A So, M Hofer

**Affiliations:** 1 CHUV / Rhumatology, Lausanne, Switzerland; 2 CHUV / Pediatrics, Lausanne, Switzerland; 3 HUG / Service of Genetic Medicine, Geneva, Switzerland

## Introduction

PFAPA is a pediatric auto-inflammatory syndrome of unknown etiology, characterized by recurrent fever, aphthosis, pharyngitis and cervical adenitis. Dysregulated monocyte interleukin-1 beta (IL-1β) secretion is thought to play an important role in fever flares. Recently, it was published that Thp1 cells (a monocytic cell line) transduced with the Q705K variant of NLRP3 increased IL-1β secretion after alum (an adjuvant used in vaccines) stimulation.

## Objectives

We hypothesized that monocytes isolated from healthy adults carrying the Q705K variant of NLRP3 secrete more IL-1β than monocytes from adults with a WT NLRP3 after ultra pure lipopolysaccharide (LPSup) stimulation.

## Methods

Monocytes of six PFAPA families whereby only one of the two parents was carrying the Q705K variant were isolated by MACS and stimulated with LPSup. Levels of IL-1β, TNF-α and IL-6 produced by monocytes isolated from Q705K positive parents were compared to family members expressing WT NLRP3.

## Results

The production of IL-1β, TNF-α or IL-6 is not significantly different between monocytes from Q705K positive and WT NLRP3 parents (Q705K NLRP3: 4583.7 ± 2671.1, 3110 ± 2904.6, and 49043.7 ± 37257.9 pg/ml; WT NLRP3: 3499.4 ± 2946.7, 935.6 ± 1259.4, and 45982 ± 18317.4 pg/ml respectively).

## Conclusion

Our results show that the Q705K variant of NLRP3 do not lead to any modulation in cytokine production capacity following LPSup stimulation, as compared to WT controls.

## Disclosure of interest

None declared

